# Temporal trends in malignancy incidence and outcomes among kidney transplantation recipients: a multi-center real-world evidence study using the TriNetX network (2000–2010 vs. 2010–2021)

**DOI:** 10.3389/fimmu.2025.1626135

**Published:** 2025-10-02

**Authors:** Gu-Shun Lai, Jian-Ri Li, Chuan-Shu Chen, Shian-Shiang Wang, Chia-Yen Lin, Che-Jui Yang, Hao-Chung Ho, Sheng-Chun Hung, Kun-Yuan Chiu, Cheng-Kuang Yang

**Affiliations:** ^1^ Institute of Medicine, Chung Shan Medical University, Taichung, Taiwan; ^2^ Department of Urology, Taichung Veterans General Hospital, Taichung, Taiwan; ^3^ Department of Medicine and Nursing, Hungkuang University, Taichung, Taiwan; ^4^ Department of Post-Baccalaureate Medicine, College of Medicine, National Chung Hsing University, Taichung, Taiwan; ^5^ Department of Applied Chemistry, National Chi Nan University, Nantou, Taiwan; ^6^ College of Medicine, National Yang Ming Chiao Tung University, Taipei, Taiwan; ^7^ Jenteh Junior College of Medicine, Nursing and Management, Miaoli, Taiwan

**Keywords:** cancer, kidney transplantation, outcomes, trend, maligancy

## Abstract

**Abbreviations:**

BMI, body mass index; CI, confidence interval; ESRD, end stage renal disease; HR, hazard ratio; IRB, Institutional Review Board; ICD, International Classification of Diseases; KTX, kidney transplantation; mTOR, mammalian target of rapamycin; PSM, propensity scoring matching; PTM: post-transplantation malignancy; SIR, standardized incidence ratio; SMR, standardized mortality rate.

**Objective:**

Malignancy is a main cause of mortality and morbidity in kidney transplantation recipients. Advancements in cancer surveillance and treatment may contribute to increased incidence and improved clinical outcomes. This study aimed to investigate the trends and clinical outcomes of post-transplantation malignancies over the past two decades.

**Methods:**

We conducted a retrospective cohort study using the TriNetX network. Common post-transplantation malignancies were identified, and outcomes were assessed using Kaplan–Meier survival analysis with propensity score matching. We compared two transplantation cohorts (2000–2010 and 2011–2021) to assess potential changes in malignancy incidence, graft survival, and patient mortality.

**Results:**

A total of 184,267 kidney transplantation recipients were included. Compared to the general population, transplantation recipients exhibited a higher risk of malignancy [standardized incidence ratio (SIR) 1.635; 95% confidence interval (CI), 1.600–1.670] and mortality [hazard ratio (HR) 1.115; 95% CI, 1.071–1.763; *P* < 0.0001]. The overall incidence of post-transplantation malignancies remained stable over the past two decades. Significant reductions in graft failure (HR 0.442; 95% CI, 0.413–0.473; *P* < 0.0001) and all-cause mortality (HR 0.755; 95% CI, 0.713–0.801; *P* < 0.0001) were observed in the recent decade.

**Conclusion:**

Kidney transplantation recipients remain at increased risk for malignancies and associated mortality. While the incidence of malignancies has not changed significantly over the past two decades, both graft failure and mortality have declined in the recent decade, potentially reflecting improvements in post-transplantation care and cancer management.

## Introduction

Kidney transplantation (KTX) is a standard treatment for patients with end stage renal disease (ESRD). For patients under dialysis, KTX prolonged overall survival (OS) and improved life quality (1, 2). Malignancy, infection and cardiovascular disease are the leading causes of mortality and morbidity in KTX recipients (3–7).

KTX recipients have a cancer incidence rate approximately twofold to fourfold higher than the age- and gender- matched general population. The main mechanisms of an elevated malignancy incidence rate are likely the long-term immunosuppress agents use, carcinogenic virus infection, and donor transmission (8–17). Immunosuppressants use with an increasing risk of carcinogenic virus infection may be the cause of post-transplantation cancer occurrence, such as liver cancer caused by hepatitis B virus. Risk increased for common cancers in the general population, for example, skin, lung and kidney cancer, which may be attributable to multi-factors, including immunosuppression, carcinogenic medication, or end stage renal disease (18–20). Certain cancer types (breast and prostate cancer) have decreased incidence after kidney transplantation for unknown reasons (12, 13).

Improvement in post-kidney transplantation care results in a longer graft survival and a reduction in recipient mortality caused by infection or cardiovascular events (3–7). Meanwhile, a longer survival may contribute to an increased incidence of post-transplantation malignancy. Despite recent advancement in cancer management, it is challenging in managing transplantation recipients with cancer when preserving renal function at the same time (21–28).

In view of the increased risk of post-kidney transplantation malignancy and improved cancer treatment, we conducted here a cohort study to evaluate the variation in cancer incidence and outcomes for kidney transplantation recipients in the past two decades.

## Materials and methods

### Data source and study population

We conducted a retrospective cohort study on the TriNetX network, a database with more than 275 million population around the world. The TriNetX platform provided real-world and updated data on demographics, diagnosis, laboratory values, medications and procedures. Within the database, we used the US Collaborative Network, which is a sub-database of the network including 57 US healthcare organizations. Data retrieval and analysis were carried out in June, 2024.

We included patients aged ≥18 years who had received kidney transplantation during the period from January 1, 2000 to December 31, 2021. Patients with kidney transplantation were included and identified using the International Classification of Diseases, 10th edition, Clinical Modification (ICD-10-CM): ICD-10-CM Z94.0, as well as other ICD-10-CM codes to confirm the diagnosis of cancer (**Supplementary Data 1**). The index date was set at the date of kidney transplantation when evaluating time from transplantation to cancer diagnosis, and cancer diagnosis when calculating graft failure and overall mortality. To ensure the inclusion of all post-transplant cancer cases while minimizing discrepancies in registration timing in the database, post-transplantation malignancy (PTM) was defined as a diagnosis made at least one month following the transplantation. Recipients with cancer diagnosis before KTX were excluded, since our aim was to investigate *de novo* malignancy after KTX.

### Statistical analyses

For baseline characteristics, mean and standard deviation were used to represent continuous variables, and number (percentage) to represent categorical variables. To evaluate the risk of specific malignancy in KTX recipients, we evaluated the standardized incidence ratios (SIR) between recipients and the general population after adjusted with age, gender (male/female) and race (Caucasian/African American/Asian). When calculating SIR, the general population was identified using ICD-10: Z00.0 (encounter for general adult medical examination) and patients with history of transplantation were excluded. Kaplan-Meier method with propensity score matching (PSM) was used to evaluate the relative risk of mortality among patients with or without PTM, and the incidence and outcomes of PTM between two different periods (2000–2010 and 2011-2021). To evaluate relative mortality, the control cohorts (the general population with cancer) were identified using corresponding ICD codes of malignancies, excluding patients with history of transplantation. For the trends over the past two decades, we compared the overall mortality and graft failure (ICD-10-CM: T86.12) between the two cohorts (2000–2010 versus 2011-2021), using 2000–2010 as a reference cohort. We performed all the statistical analyses on the platform. Statistically significance was set at P <0.05.

### Ethics in research

Our study was carried out after approval from the Institutional Review Board (IRB) of Taichung Veterans General Hospital (IRB number: SE:22220A). Since all data on the database was de-identified, informed consent was waived by the ethics committee.

## Results

### Patient characteristics

We studied a total of 184,267 patients who had received kidney transplantation in the period between 2000 and 2021. During the follow-up period, 30, 545 (16%) patients had developed *de novo* malignancy after kidney transplantation. When compared with those without cancer, patients who had developed cancer after kidney transplantation were older, with more male in gender, more white patients, higher body mass index (BMI), higher rates of rejection, smoking, comorbidities; they also had lower incomes, and lower serum Tacrolimus level (All P<0.0001). **Table 1** shows the patients baseline demographics.

**Table 1:** Baseline characteristics for patients with or without post-kidney transplantation malignancy.

	KTX with cancer n = 30,545	KTX without cancer n=153,722	P
Age at KTX, years	56.8 (12.9)	51.8 (15.5)	<0.0001
Age at cancer diagnosis, years	60.3 (12.6)	none	
Male Gender, n (%)	16209 (57.9)	78513 (54.4)	<0.0001
Race, n (%)			
White patients	17857 (64)	72104 (49.9)	<0.0001
African American	4133 (15)	31935 (22.1)	<0.0001
Asian patients	915 (3)	7460 (5)	<0.0001
others/unknown	7640 (25)	37937 (25)	<0.0001
History of rejection, n (%)	9160 (33)	30721 (21)	<0.0001
Rejection before cancer, n (%)	7230 (24)	23490 (16.2)	<0.0001
Serum Tacrolimus level, ng/ml	5.94 (3.25)	6.52 (3.4)	<0.0001
Serum Tacrolimus level before cancer, ng/ml*	6.4 (3.36)	6.77 (3.3)	<0.0001
Induction therapies, n (%)			
Basiliximab	666 (3)	1699 (1)	<0.0001
Anti-thymocyte globulin	515 (2)	3058 (2)	0.2
Maintenance Immunosuppression, n (%)			
Tacrolimus	19328 (68)	84900 (57)	<0.0001
Cyclosporin	3357 (12)	8884 (6)	<0.0001
Mycophenolate mofetil	14712 (52)	62851 (42)	<0.0001
mTOR inhibitor	4101 (14)	6841 (5)	<0.0001
Prednisolone	20764 (73)	84590 (57)	<0.0001
Comorbidities, n (%)			
Hypertension	15901 (56)	76408 (51)	<0.0001
Diabetes Mellitus	8822 (31)	42472 (29)	<0.0001
Insulin injection	6695 (24)	32204(22)	<0.0001
Ischemic heart disease	6303 (22)	25946 (17)	<0.0001
Cerebrovascular disease	3207 (11)	12027 (8)	<0.0001
Body Mass Index, n (%)	28.3(5.97)	28.3 (6.09)	0.86
Smoking, n (%)	3687 (13)	14840 (10)	<0.0001
Knorfsky performance status	84.4 (15.3)	85.3 (16.4)	0.59

KTX, kidney transplantation; mTOR, mammalian target of rapamycin.

*Calculated at 2.54 years after KTX for patients without malignancy.

### Incidence of various cancers

Common types of cancer that had occurred following kidney transplantation are listed on **Table 2**. The most common PTM was non-melanoma skin cancer (31.4%), followed by kidney cancer (9.3%), prostate cancer (6.8%), leukemia (6.6%), non-Hodgkin lymphoma (NHL) (6.2%), lung cancer (5.3%), breast cancer (3.9%), liver cancer (3.2%), colon cancer (2.9%), and bladder cancer (2.8%). Median age at cancer diagnosis was 60.3 years. Patients with NHL (52 years old for KTX and 55.9 cancer diagnosis) were younger at both KTX and the diagnosis of cancer, while patients developing lung cancer (61 for KTX and 65 for cancer diagnosis) and prostate cancer (62.7 for KTX and 66.4 years for cancer diagnosis) were older. Median time from KTX to cancer diagnosis was 2.54 years, with colon cancer having the longest time (3.3 years), and liver cancer (2.16 years) having the shortest time.

**Table 2:** Malignancy distribution, incidence and outcomes in kidney transplantation recipients.

	Number (%)	Male, n (%)	Age at KTX, years	Age at cancer diagnosis, years (SD)	Median time to cancer, years (SD)	SIR (95% CI)^a^	P	Mortality HR (95% CI)^b^	P
Non-melanoma skin	9611 (31.4)	6301 (65.5)	59.2 (10.8)	63.1 (10.4)	3	2.346 (2.262-2.433)	<0.0001	2.882 (2.699-3.007)	<0.0001
Kidney	2841 (9.3)	1902 (67)	55 (13.4)	58.6 (13.1)	2.39	5.199 (4.785-5.68)	<0.001	1.146 (1.049-1.251)	0.0024
Prostate	2076 (6.8)	1969 (100)	62.7 (9.7)	66.4 (9.15)	2.75	0.764 (0.722-0.809)	<0.0001	1.53 (1.3-1.799)	<0.0001
Leukemia	2007 (6.6)	1208 (60)	55 (14.5)	58.6 (14.5)	2.72	2.376 (2.18-2.556)	<0.0001	1.218 (1.105-1.344)	<0.0001
Non-Hodgkin lymphoma	1906 (6.2)	1140 (59.8)	52.1 (16.1)	55.9 (16.1)	2.73	2.818 (2.579-3.079)	<0.0001	1.942 (1.734-2.174)	<0.0001
Lung	1621 (5.3)	1011 (62.3)	61 (10.5)	65 (10.3)	3.25	1.138 (1.06-1.222)	0.0003	1.409 (1.283-1.543)	<0.0001
Breast	1181 (3.9)	67 (5.6)	56.6 (11.6)	60.7 (11.2)	2.18	0.746 (0.69-0.808)	<0.0001	2.17 (1.852-2.543)	<0.0001
Liver	965 (3.2)	578 (64)	56.8 (12.3)	60.3 (12.2)	2.16	2.043 (1.82-2.293)	<0.0001	0.407 (0.371-0.446)	<0.0001
Colon	898 (2.9)	506 (56.3)	57.4 (13.3)	61.7 (12.8)	3.36	1.177 (1.063-1.304)	0.0003	1.653 (1.42-1.925)	<0.0001
Bladder	840 (2.8)	567 (67.9)	60.3 (12.8)	63.9 (12.7)	2.5	1.291 (1.16-1.438)	<0.0001	1.778 (1.552-2.078)	<0.0001
All types of cancer	30,545	16209 (57.9)	56.8 (12.9)	60.3 (12.6)	2.54	1.635 (1.6-1.67)	<0.0001	1.115 (1.071-1.163)	<0.0001

CI, confidence interval; HR, hazard ratio; KTX, kidney transplantation; SD, standard deviation; SIR, standardized incidence ratio.

a Compare with the general population, adjusted with age, gender, and race.

b Compare with the general population with cancer, adjusted with age, gender, and race.

We further compared the cancer incidence between KTX patients and the general population after PSM. After PSM for age, gender and race, patients receiving KTX were associated with a higher risk for cancer development compared with the general population [SIR 1.635, 95% confidence interval (CI) 1.6-1.67]. In general, KTX patients had a higher risk for cancer occurrence for most of the cancer types. Among different sites of cancer, the highest risk of PTM was kidney cancer (SIR 5.199, 95% CI 4.785-5.68), followed by NHL (SIR 2.818, 95% CI 2.579-3.079) and leukemia (SIR 2.376, 95% CI 2.18-2.556).

### Risk factors for malignancy

**Figure 1:**
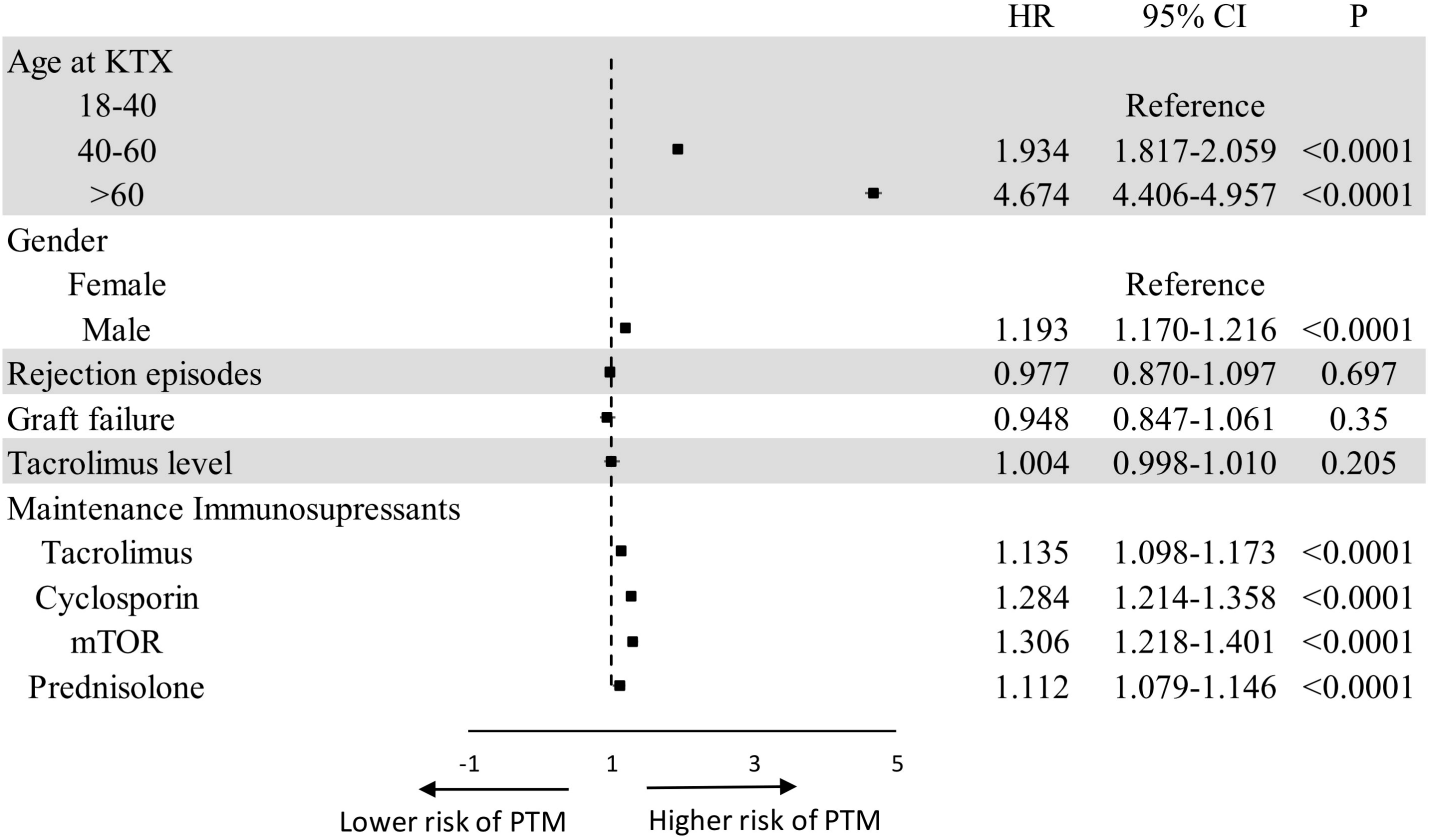
Forest plot of multivariate analysis on risk factors for PTM. CI, confidence interval; HR, hazard ratio; KTX, kidney transplantation; OS, overall survival; PTM, post-transplant malignancy.

Through the multivariate cox regression analysis, factors revealed for PTM were as follows: older age (40–60 years HR 1.934 95% CI 1.817-2.059 P<0.0001, > 60 years HR 4.674 95% CI 4.406-4.957 P<0.0001), male gender (HR 1.193 95% CI 1.170-1.126 P<0.0001), tacrolimus (HR 1.135 95% CI 1.098-1.173 P<0.0001), cyclosporin (HR 1.284 95% CI 1.214-1.358 P<0.0001), mTOR (HR 1.306 95% CI 1.218-1.401 P<0.0001), and prednisolone (HR 1.112 95% CI 1.079-0.1.146 P<0.0001). **Figure 1** presents the results of the multivariate analysis and forest plot on factors for PTM in kidney transplantation recipients.

### Trends for post-KTX malignancy

A total of 56,922 patients who received KTX in 2000–2010 and 127,345 patients in 2011–2021 were included. During the follow-up period, 12,921 (22.7%) patients in the 2000–2010 cohort and 15,026 (11.8%) in the 2011–2021 cohort experienced PTM. After adjusted for age, gender, and race, there was no change in overall cancer incidence over the past two decades (HR 1.003 95% CI 0.956-1.053, P = 0.892). Regarding the specific site of cancer, there was a decrease in incidence for leukemia (HR 0.615 95% CI 0.518-0.729, P < 0.0001) and bladder cancer (HR 0.741 95% CI 0.576-0.954, P = 0.019). **Table 3** shows the unadjusted and adjusted incidence hazard ratios of PTM between the 2000–2010 and 2011–2021 cohorts.

**Table 3:** Unadjusted and adjusted incidence hazard ratios for post-transplantation malignancy between patients receiving kidney transplantation at 2000-2010 or 2011-2021, using 2000-2010 cohort as a reference.

	Unadjusted			Adjusted^a^		
	HR	95% CI	P	HR	95% CI	P
Non-melanoma skin	1.036	0.985-1.089	0.17	0.973	0.905-1.047	0.463
Kidney	1.312	1.203-1.432	<0.0001	1.04	0.897-1.206	0.605
Prostate	1.285	1.16-1.424	<0.0001	1.005	0.837-1.207	0.956
Leukemia	0.639	0.547-0.713	< 0.0001	0.615	0.518-0.729	< 0.0001
Non-Hodgkin lymphoma	1.048	0.926-1.186	0.457	0.981	0.818-1.176	0.835
Lung	1.14	1.006-1.292	0.04	1.028	0.858-1.232	0.765
Breast	1.013	0.871-1.179	0.863	0.848	0.675-1.066	0.157
Liver	1.252	1.046-1.499	0.014	1.079	0.835-1.396	0.56
Colon	1.086	0.922-1.279	0.322	0.976	0.768-1.239	0.848
Bladder	1.047	0.892-1.229	0.573	0.741	0.576-0.954	0.019
All types of cancer	1.103	1.069-1.139	<0.0001	1.003	0.956-1.053	0.892

CI, confidence interval; HR, hazard ratio.

^*^Adjusted for age, gender and race.

**Figure 2:**
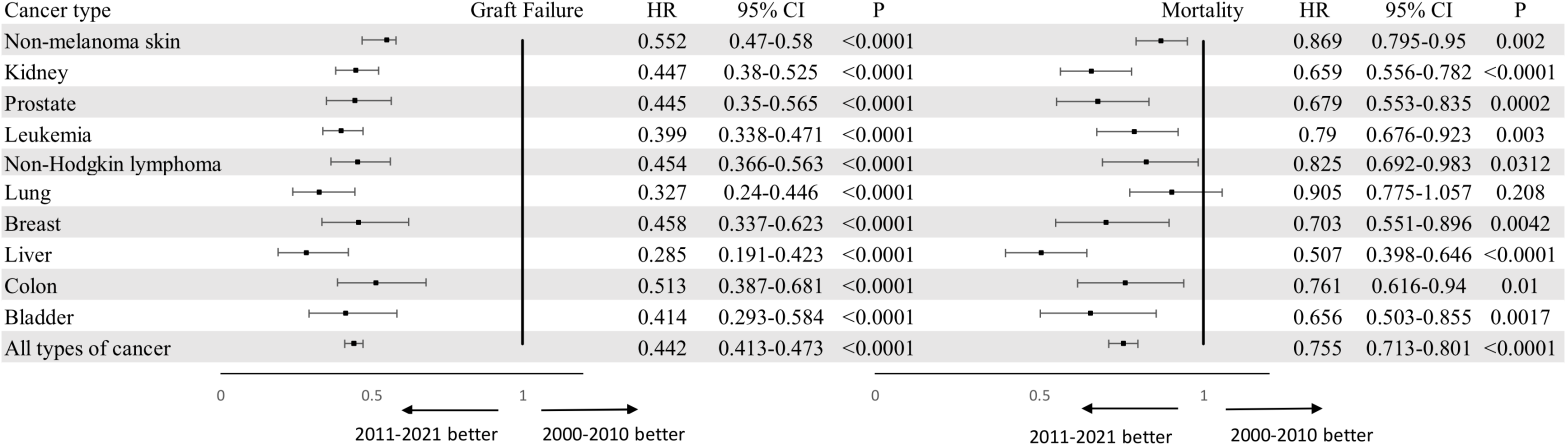
Forest plots of graft failure and overall mortality for kidney transplantation recipients with malignancy who underwent KTX in 2000-2010 or 2011-2021 using 2000-2010 cohort as a reference. Adjusted for age, gender and race. KTX, kidney transplantation.

**Figure 3:**
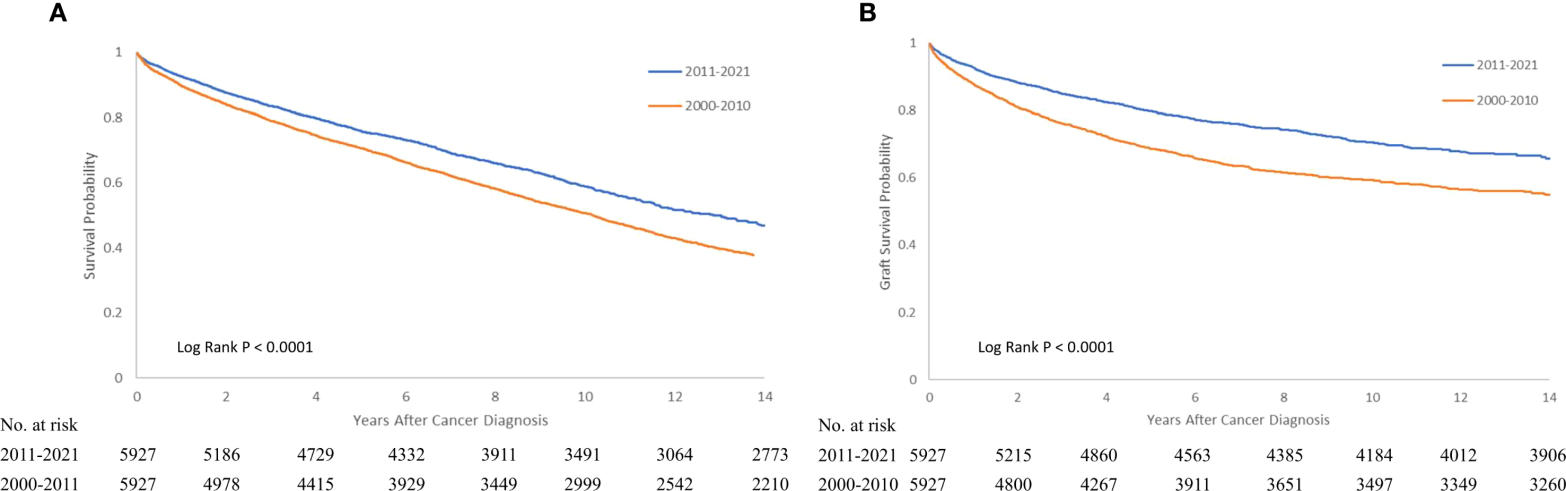
Kaplan-Meier survival curve of overall **(A)** and graft survival **(B)** for recipients with post-KTX malignancy who underwent KTX in 2000-2010 or 2011-2021. KTX, kidney transplantation.

Regarding the variation of outcomes (graft failure and mortality) for patients with PTM over the past two decades, we found a trend toward reduction in graft failure (HR 0.442 95% CI 0.413-0.473, P < 0.000) and overall mortality (HR 0.755 95% CI 0.713-0.801, P < 0.0001 for overall mortality). **Figures 2**, **3** compares the graft failure and mortality rates for post-KTX malignancy between the 2000–2010 and 2011–2021 cohorts.

### Impact of malignancy on outcomes for KTX recipients

Patients with PTM were associated with a higher risk of mortality (HR 1.115 95% CI 1.071-1.763 P < 0.0001) compared with the general population with malignancy after PSM. For specific cancer: non-melanoma skin cancer (HR 2.88 95% CI 2.699-3.007) and breast cancer (HR 2.17 95% CI 1.852-2.543) were associated with the worst overall survival compared with the general population with cancers (**Table 2**).

## Discussion

In this study, we found that patients with KTX were associated with a higher risk of malignancy when compared with the general population. Recipients with post-KTX malignancy had worse overall survival compared with the general population with malignancy. There was no significant change in cancer incidence for KTX recipients over the past 20 years, but graft failure and mortality rates decreased in the recent decade.

Previous studies reported higher malignancy risks of 2 to 4-fold in kidney transplantation recipients compared with the general population (8–17). In this study, we found that such patients had an increased risk of developing malignancy following kidney transplantation (SIR:1.635) compared with the general population. The slightly lower SIR is likely related to the fact that we excluded patients with history of malignancy before transplantation. In general, most sites of cancer presented higher SIR compared with the general population. On the other hand, breast and prostate cancer did not show an elevated risk, the real reason was unclear, and this finding was consistent with the study conducted by Engels et al. (12).

A higher risk of malignancy for KTX recipients may be resulted from several factors (16). Immunosuppression may be the reason for virus induced cancers. For example, hepatitis B virus and hepatitis c virus for liver cancer, and human T cell lymphotropic virus for non-Hodgkin lymphoma. On the other hand, for non-virus mediated cancers, including common cancers in general population (lung, colon, breast, and prostate cancer) and ESRD patients (kidney and bladder cancer), closer cancer screening tests may have contributed to a higher incidence rate for those patients (18–20).

The mortality rates between KTX patients with cancer and the general population with cancer were debatable. Kiberd et al. reported that there was no difference in cancer mortality between these two populations (29). However, some studies report a higher mortality rate for patients with PTM compared with the general population (30–32). In this study, we found that the overall survival of KTX recipients was worse for those with PTM compared with the general population for most cancer types and overall malignancy (HR 1.115 95% CI 1.071-1.763 P < 0.0001). The results indicated a challenging condition in treating these KTX recipients with malignancy under immunosuppressants, which may limit the use of chemotherapy or immunotherapy.

Previous reports on the median time from kidney transplantation (KTX) to cancer diagnosis have been limited and variable, ranging from 2.6 to 4 years (32, 33). In our study, the interval from KTX to cancer diagnosis was comparatively shorter (2.54 years). This difference may be attributable to more intensive cancer screening following KTX as well as the increasing use of marginal donors and recipient. Importantly, these results highlight the need for closer surveillance for common post-transplant malignancies.

The use of mammalian target of rapamycin (mTOR) inhibitors has been associated with a reduced incidence of malignancy in several studies (28, 34, 35). However, conflicting evidence has been reported regarding the cancer risk associated with sirolimus use in recipients (36, 37). A meta-analysis by Knoll et al. indicated that the anti-cancer benefits of sirolimus were primarily observed in patients who were converted from other immunosuppressive regimens, rather than in those who received *de novo* sirolimus (34). Furthermore, *de novo* use of sirolimus was associated with an increased risk of post-transplant lymphoproliferative disorder (38). In our study, multivariate analysis revealed that the use of mTOR inhibitors was associated with an increased risk of PTM (HR 1.306–95 CI 1.218-1.401). Previous studies have shown that calcineurin inhibitors (CNIs), such as cyclosporin and tacrolimus, are associated with a heightened risk of PTM, potentially due to their immunosuppressive or carcinogenic properties (32, 39). Consistent with these findings, multivariate analysis identified tacrolimus (HR 1.135–95 CI 1.098-1.173) and cyclosporin (HR 1.284–95 CI 1.214-1.358) as risk factors for PTM when used as maintenance immunosuppressive therapies. Notably, we did not observe a significant association between elevated serum tacrolimus levels and PTM. This may be attributed to the dynamic nature of tacrolimus serum levels and the lack of standardized timing for level measurement in the TriNetX database. These findings underscore that immunosuppression remains a key factor for PTM, regardless of the specific regimen used. Therefore, enhanced cancer surveillance is warranted in kidney transplant recipients (24, 40, 41).

Blosser CD, et al. and Piselli P, et al. reported that there was no significant change in incidence of PTM over time (42, 43). Our results align with previous evidence, indicating that the overall incidence of PTM has remained relatively stable over the last twenty years. In the unadjusted analysis, increased incidences of kidney, prostate, and overall cancer were observed. These findings may be attributable to the older age of recipients and the increased intensity of cancer screening in recent years. However, after adjusting for these factors, the overall cancer incidence did not show a significant increase over the past two decades. Blosser et al. previously reported no significant improvement in graft or patient survival among kidney transplant recipients with non-Hodgkin lymphoma (non-NHL) malignancies (42). In contrast, our study demonstrated a decline in both graft failure and patient mortality among recipients diagnosed with PTM over the past two decades. Although the exact mechanisms underlying these observations remain unclear, the trends may be partially attributable to improvements in post-transplantation care, including advancements in oncologic treatments and the management of infectious and cardiovascular complications, which could positively influence graft and patient survival.

This study has several limitations inherent to its retrospective design, including the possibility of residual confounding despite adjustment for known covariates. The use of the TriNetX database, while providing a large sample size, is limited by the absence of detailed cancer-specific outcome metrics such as tumor staging, histopathology, treatment modalities, and cause-specific mortality, which restricts the depth of interpretation. Additionally, the reliance on administrative coding may introduce misclassification bias in diagnoses and outcomes. Selection bias is also a concern, as the dataset represents patients from specific healthcare organizations and may not fully generalize to the broader transplant population. Furthermore, patients in the more recent transplant cohort may have shorter follow-up durations, potentially leading to an underestimation of cancer incidence in this group. Lastly, we defined PTM as any cancer diagnosis occurring ≥1 month following kidney transplantation. It is acknowledged that this definition may inadvertently capture malignancies that were present but undiagnosed at the time of transplantation. Despite these limitations, our research was a population-based study with a large sample size, which may avoid some bias arising from its retrospective nature. Furthermore, data regarding the trends of PTM over the past decades was limited, and our research provided information on this topic.

In conclusion, KTX recipients exhibited a significantly higher risk of PTM compared with the general population. Moreover, the presence of PTM was associated with increased overall mortality. Although the incidence of PTM has remained stable over the past two decades, both graft failure and mortality risks among patients with PTM have shown a significant decline during the same period.

## Data Availability

The original contributions presented in the study are included in the article/**Supplementary Material**. Further inquiries can be directed to the corresponding author.
